# Fbxw7-associated drug resistance is reversed by induction of terminal differentiation in murine intestinal organoid culture

**DOI:** 10.1038/mtm.2016.24

**Published:** 2016-04-13

**Authors:** Federica Lorenzi, Roya Babaei-Jadidi, Jonathan Sheard, Bradley Spencer-Dene, Abdolrahman S Nateri

**Affiliations:** 1 Cancer Genetics and Stem Cell Group, Cancer Biology Unit, Division of Cancer and Stem Cells, School of Medicine, University of Nottingham, Nottingham, UK; 2 CM Technologies Oy I, Institute for Biomedical Technology, University of Tampere, Tampere, Finland; 3 Experimental Pathology Laboratory, Cancer Research UK London Research Institute, The Francis Crick Institute, Lincoln’s Inn Fields Laboratory, London, UK

## Abstract

Colorectal cancer (CRC) is one of the top three cancer-related causes of death worldwide. FBXW7 is a known tumor-suppressor gene, commonly mutated in CRC and in a variety of other epithelial tumors. Low expression of FBXW7 is also associated with poor prognosis. Loss of FBXW7 sensitizes cancer cells to certain drugs, while making them more resistant to other types of chemotherapies. However, is not fully understood how epithelial cells within normal gut and primary tumors respond to potential cancer therapeutics. We have studied genetically engineered mice in which the *fbxw*7 gene is conditionally knocked-out in the intestine (*fbxw*7^∆G^). To further investigate the mechanism of Fbxw7-action, we grew intestinal crypts from floxed-*fbxw*7 (*fbxw*7^fl/fl^) and *fbxw*7^ΔG^ mice, in a Matrigel-based organoid (mini-gut) culture. The *fbxw*7^ΔG^ organoids exhibited rapid budding events in the crypt region. Furthermore, to test organoids for drug response, we exposed day 3 intestinal organoids from *fbxw*7^fl/fl^ and *fbxw*7^∆G^ mice, to various concentrations of 5-fluorouracil (5-FU) for 72 hours. 5-FU triggers phenotypic differences in organoids including changing shape, survival, resistance, and death. 5-FU however, rescues the drug-resistance phenotype of *fbxw*7^ΔG^ through the induction of terminal differentiation. Our results support the hypothesis that a differentiating therapy successfully targets FBXW7-mutated CRC cells.

## Introduction

Common anticancer strategies often fail resulting in tumor recurrences and metastasis. Only about 6% of colorectal cancer (CRC) metastatic patients live more than 5 years after diagnosis.^1^ There is an urgent need to develop molecularly targeted cancer therapies and drug-screening models to stratify patients and achieve maximum benefit from expensive treatments. The recent advent of organoid (mini-gut) culture has allowed development of an *in vitro* model that mimics many aspects of colon/intestinal homoeostasis and tumors.^2–7^ Sato and colleagues,^7^ initially established the ideal conditions for isolated small intestinal crypts or single leucine-rich repeat-containing G-protein-coupled receptor 5 (Lgr5),^8^ positive stem cells to develop into organoids: laminin-rich Matrigel mimicking the basal lamina and a finely balanced medium including R-spondin1 (Wnt agonist),^9^ Noggin (BMP antagonist)^10^ and epidermal growth factor,^11^ were found sufficient to maintain the proliferation of the stem cell compartment or single Lgr5-positive cells. Similar to small intestine, the stem cell compartment at the base of the crypt-like domain of the mini-guts maintains epithelial turnover: stem cells give rise to progenitor cells, which differentiate while migrating toward the villus-like domain into enterocytes, enteroendocrine cells, and goblet cells. Paneth cells, instead, are located at the bottom of the crypt. In the organoid model, tumor cells can grow in a more similar manner to that in living organisms, with cell-cell interactions and boundaries. The modeling of cancer mutations in human intestinal/colon organoids using new tools for gene manipulation, allowed both *in vitro* and *in vivo* experimental approaches for mutational and functional analysis.^3–5^ Organoid culture can be used indefinitely. Moreover, changes in the composition of the medium can drive changes in phenotype/structure of organoids that facilitates *in vitro* testing of drugs, inhibitors, and growth factors. This model may also present a novel method of drug screening.^4,12^

One of the key cellular mechanisms of regulating activity of transcriptional-activators is through protein degradation using the ubiquitin-proteasome system. Specificity of proteolysis for any particular substrate is determined by its association with a specific E3-receptor subunit. FBXW7 (also called hCDC4, Ago, Sel10, and Fbw7) functions as a receptor subunit for the Skp1/Cullin/F-box (SCF)-E3-ubiquitin-ligase (SCF^FBXW7^). Recent excellent reviews on FBXW7 confirm that loss of FBXW7 function is likely to result in failed regulation of its downstream proteins targets, which disrupt a variety of critical signaling pathways resulting in acquisition of the hallmarks of cancer.^13–20^ Underlining the importance of FBXW7 as a tumor suppressor is the fact that loss of function mutations in the FBXW7 gene occur in a variety of human cancers including 10–15% of CRCs.^21–23^

We and others have previously studied the Fbxw7 conditional knockout in murine intestinal lineages and cancer.^24,25^ The synergistic contribution of FBXW7 and TP53 to the suppression of gastrointestinal cancer has also been reported.^26,27^ Interestingly, an intestinal knock-in model of Fbxw7 (R482Q) mutation has recently been described,^28^ nevertheless both Fbxw7 conditional knockout and knock-in models promote an equivalent intestinal tumorigenesis on an *adenomatous polyposis coli* (*Apc*) mutant background.^25,28^ FBXW7 mutations have been investigated comprehensively in CRC.^23,29^ Understanding FBXW7 loss-associated mechanism(s) driving drug resistance could therefore be important to successfully target and eradicate CRC cells carrying FBXW7 mutations. Recent literature has reported FBXW7 loss confers resistance to oxaliplatin and cisplatin chemotherapeutic agents,^30,31^ while CRC cell lines harboring FBXW7 mutations or deletions are sensitive to Rapamycin treatment.^32^

Here, we employed the murine intestinal organoid system, to identify cellular response mediated by Fbxw7 to various concentrations of 5-fluorouracil (5-FU), a DNA damage agent commonly administered to metastatic CRC patients.^1^ We show that *fbxw*7 gut-specific inactivation (*fbxw*7^ΔG^) with high proliferative capacity give rise to microadenoma-like structures. 5-FU promotes terminal differentiation and apoptosis in control floxed-*fbxw*7 (*fbxw*7^fl/fl^) organoids. Intestinal organoids from *fbxw*7^∆G^ are two to three times less sensitive to 5-FU treatment than *fbxw*7^fl/fl^ intestinal organoids. Moreover, epithelial cells extravasated from the *fbxw*7^∆G^ organoids were abrogated by the 5-FU treatment, and the induction of cell differentiation.

## Results

### FBXW7 loss in human CRC cells promotes resistance to 5-FU

**Figure 1:**
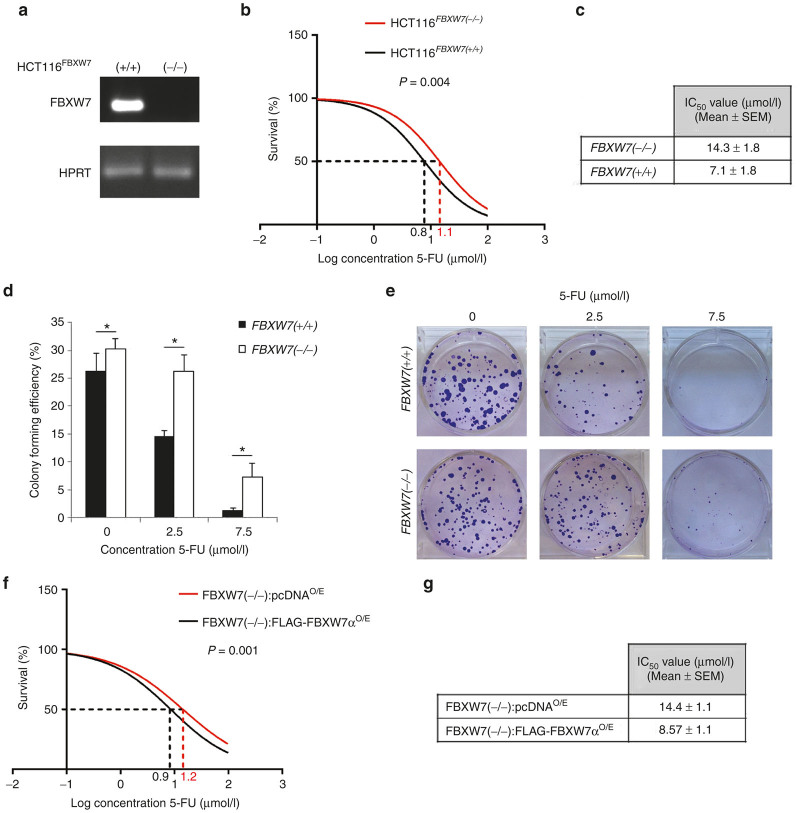
*FBXW*7 deletion confers 5-FU resistance to human colon cancer HCT116 cells. (**a**) RT-PCR shows *FBXW7* status in HCT116^FBXW7(+/+)^ and HCT116^FBXW7(−/−)^ cell lines. Reverse primer was designed to target the deleted exon 5 of *FBXW7*. (**b,c**) Sulforhodamine B (SRB) assay to test the cytotoxicity of 5-FU in both HCT116^FBXW7(+/+)^ and HCT116^FBXW7(−/−)^ cell lines. Vehicle (dimethyl sulfoxide) and 10 doses of 5-FU (from 0.05 to 100 μmol/l) were incubated for 72 hours following cell synchronization. (**d,e**) Colony-forming efficiency assay of HCT116^FBXW7(+/+)^ and HCT116^FBXW7(−/−)^ cell lines. 0, 2.5, and 7.5 μmol/l 5-FU was replaced after 72 hours incubation with fresh medium. At day 10 of growth, colonies were stained with crystal violet and counted by ImageJ. (**f,g**) Cytotoxicity of 5-FU was analyzed via SRB assay following transient transfection of HCT116^FBXW7(−/−)^ cells with pcDNA3 (control) or FLAG-FBXW7α plasmids as previously described.^30^ IC50 values, indicated in the table, were calculated via GraphPad Prism software. Experiments were performed in triplicate and repeated at least in three independent occasions. Data are mean ± standard error of the mean (*P < 0.05, **P < 0.01, ***P < 0.001).

The chemotherapeutic agent 5-FU is used to treat many different types of cancer, including gastrointestinal cancers. *In vitro*, 5-FU can trigger apoptosis in CRC cells and it appeared to be initiated by caspase-dependent death pathway.^33,34^ To investigate the role of FBXW7 mutation in drug resistance, we examined the induction of growth inhibition in a dose-dependent manner using FBXW7 knockout HCT116 (HCT116^FBXW7(−/−)^) and DLD-1 (DLD-1^FBXW7(−/−)^),^23,24^ in parallel with FBXW7 wild-type (FBXW7^(+/+)^) counterpart cell lines (Figure 1a and Supplementary Figure S1a). Seeded cells were synchronized overnight by serum starvation to reduce the FBXW7 deficiency-induced cell division/growth in CRC cells, as reported previously,^23,24,30,32,35^ prior to 72 hours incubation with 5-FU for cytotoxicity assay. Sulforhodamine B colorimetric assay was carried out to measure the IC_50_. IC_50_ of HCT116^FBXW7(−/−)^ cells (14.3 μmol/l) was twofold higher than the IC_50_ of HCT116^FBXW7(+/+)^ cells (7.1 μmol/l) (Figure 1b,c). In addition, the colony-forming efficiency assay revealed that HCT116^FBXW7(−/−)^ exhibited a significant resistance to 5-FU (Figure 1d,e). In accordance with previous studies using oxaliplatin and cisplatin,^30,31^ deletion of *FBXW7* mediated higher tolerance of 5-FU in HCT116 cells. To test whether re-expression of FBXW7 render CRC cells sensitive to 5-FU, as Fbxw7α was shown to be the preferentially expressed Fbxw7 isoform in intestine/colon,^24^ we have overexpressed FBXW7α in both HCT116^FBXW7(−/−)^ and DLD-1^FBXW7(−/−)^ cells. CRC cell lines are initially transfected with FLAG-FBXW7α and control pcDNA3 plasmids in 10 cm tissue culture plates. Ten hours after transfection, cells were split and seeded in 96-well plates. Transfection efficiency was determined by western blot (Supplementary Figure S1f). Cells were synchronized after overnight serum starvation, treated with increasing concentrations of the 5-FU for 72 hours and the IC50 was determined using sulforhodamine B assay as outlined above (Figure 1f,g and Supplementary Figure S1d,e). The results showed that Fbxw7α overexpression significantly decreased the 5-FU IC50 and inhibited FBXW7 mutation in mediating 5-FU drug-resistance of CRC cell lines.

### *Fbxw7*^ΔG^ organoids (mini-guts) possess a peculiar morphology

We have demonstrated intense *fbxw*7-expression in the crypt base of wild-type mice, including the transit amplifying/progenitor cells, compared with the villus using *in-situ hybridization*.^24,25^ Intestinal *fbxw*7 knockout resulted in impaired cell differentiation, with a reduction in the number of goblet and Paneth cells. However, despite the increase of actively proliferating epithelial cells in the guts of the *fbxw*7^∆G^ mice, no intestinal tumors were reported up to 11–12 months of age. This could be because—in the guts multiple signaling molecules are emerging from different linages—the underlying mesenchyme, immune and crypt-based epithelial cells may together cooperate tightly to control transformation of intestinal epithelial cells. Recently, Paneth cells were shown to be a critical component of the intestinal epithelial stem cell niche^36^; though the depletion of Paneth cells failed to alter significantly the crypt phenotype.^37^ In addition, the crypt culture recapitulates the cellular diversity, resembling proliferative and differentiated compartments of the intestinal epithelium without coculturing with stromal/fibroblast cells. Thus, to explore the function of murine epithelial Fbxw7, we cultured intestinal organoids from *fbxw7*^*fl/fl*^ and *villin-*Cre^*+*^:*fbxw7*^*fl/fl*^ (*fbxw*7^∆G^) mice (Supplementary Figure S2a,b).

**Figure 2:**
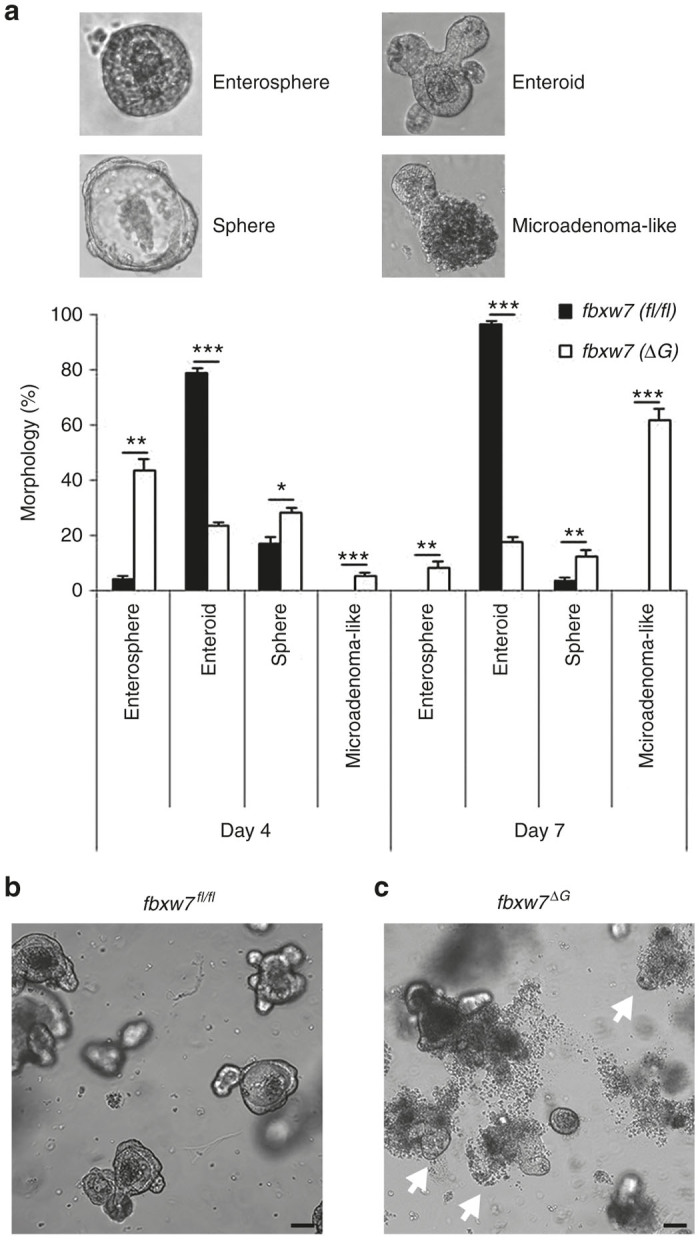
*fbxw*7-null organoids grow faster and present microadenoma-like morphology. (**a**) Percentage of enterosphere, enteroid, sphere, and microadenoma-like morphologies at day 4 and day 7 of growth of *fbxw7*^*fl/fl*^ and *fbxw7*^ΔG^ mini-guts. Mini-guts were counted by microscopy (*P < 0.05, **P < 0.01, ***P < 0.001). (**b,c**) Images are representative of *fbxw7*^*fl/fl*^ and *fbxw7*^ΔG^ mini-gut populations at day 4 of growth. Arrowheads indicate microadenoma-like mini-guts. Scale bars, 75 μm.

Small intestinal crypts isolated from *fbxw7*^*fl/fl*^ mice gave rise to enterospheres, spherical organoids formed by a monolayer of epithelial cells, within a few hours following seeding. On day 4 of growth, most of the *fbxw7*^*fl/fl*^ enterospheres were fully branched organoids (defined enteroids),^38^ and characterized by the presence of buds (Figure 2a,b). Morphology of the *fbxw7*^*fl/fl*^ organoids represent a normal intestinal organoid culture system which contains crypt and villus domains without a mesenchymal component.^7^ A low percentage of cystic organoids with an empty lumen, (defined spheres), were also present (17%). Moreover after 7 days of culture, *fbxw7*^*fl/fl*^ organoids have mainly manifested by enteroids (Figure 2a). In contrast, Fbxw7 inactivation accelerated organoid growth and altered the phenotype of intestinal organoids (Figure 2a,c). Large *fbxw7*^ΔG^ organoids were however followed by releasing clusters of cells outside the organoid lumen after 3 days of culture (Supplementary Video S1). Histologically, this peculiar morphology is highly variable in appearance, resembling a microadenoma appearance.^39^ After 7 days of culture, almost all of the *fbxw7*^ΔG^ enterospheres had changed to microadenoma-like shape (Figure 2a). Reculturing the released cells from *fbxw7*^ΔG^ organoids, demonstrated an abnormally high proliferative activity and adapted cancer cells properties (data not shown).

### Loss of Fbxw7 in intestinal organoid induces resistance to 5-FU

**Figure 3:**
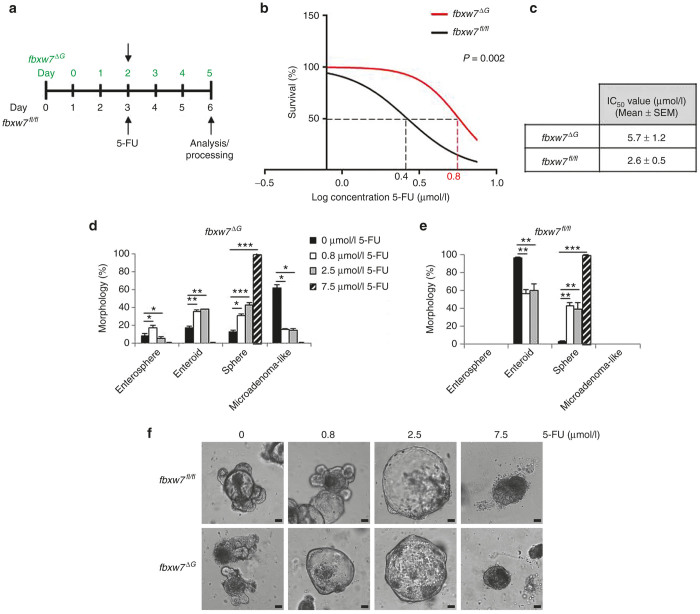
*fbxw7*^ΔG^ organoids tolerate higher dose of 5-FU. (**a**) Layout of the drug response experiments and following analysis considering faster growth of *fbxw7*^ΔG^ mini-guts. (**b,c**) Toxicity assay of 5-FU in *fbxw7*^*fl/fl*^ and *fbxw7*^ΔG^ mini-guts and IC_50_ values. (**d,e**) Morphological analysis following 72 hours incubation with 0.8, 2.5, and 7.5 μmol/l 5-FU. Percentage of enterosphere, enteroid, sphere, and microadenoma-like morphologies was assessed by microscopy in *fbxw7*^*fl/fl*^ and *fbxw7*^ΔG^ mini-gut populations. **(f)** Images represent phenotypical changes of both *fbxw7*^*fl/fl*^ and *fbxw7*^ΔG^ mini-guts in response to 5-FU. Scale bars, 50 μm. Experiments were performed in triplicate and repeated at least in two independent occasions (*P < 0.05, **P < 0.01, ***P < 0.001).

To test the loss of FBXW7 function as a potential mechanism of drug resistance, we next performed 5-FU toxicity assay in fully structured enteroids. In addition, according to a recent report,^12^ the intestinal organoids are three to four times more sensitive than CRC cell lines. Thus, both *fbxw7*^*fl/fl*^ and *fbxw7*^ΔG^ organoids were exposed to the estimated IC_50_ of HCT116^FBXW7(+/+)^ cells, 7.5 μmol/l 5-FU (Figure 1c), along with 2.5 and 0.8 μmol/l 5-FU (Figure 3a). We and other groups previously showed that the deficiency of murine intestinal Fbxw7 induced cell division via Ki67 immunohistochemistry and BrdU labelling.^24,25^ Therefore, to exclude the potential effect of cell proliferation, the 5-FU treatment started after 2 days for *fbxw7*^ΔG^ organoids, whereas for the *fbxw7*^*fl/fl*^ organoids after 3 days (Figure 3a). The toxicity assay showed that mini-guts harboring *fbxw7* deletion were approximately two to three times less sensitive to 5-FU treatment than intestinal organoids from *fbxw*7^fl/fl^ mice (Figure 3b,c), likewise HCT116^FBXW7(−/−)^ and DLD-1^FBXW7(−/−)^ cells (Figure 1b,c and Supplementary Figure S1). These results have confirmed the crucial role of Fbxw7 inactivation in the acquisition of drug resistance. Furthermore, we have compared the morphology of organoids treated with 5-FU by counting the number of viable organoids per each category; enterospheres, enteroids, spheres, and microadenoma-like structure. Quantification revealed that 5-FU promoted large spheres versus enteroids, in a dose-dependent manner, in both wild-type *fbxw7*^*fl/fl*^ and *fbxw7*^ΔG^ mutant organoids after 72 hours (Figure 3d–f). Although the highest concentration of 5-FU (7.5 μmol/l) induced the death of most of the organoids, the remaining viable organoids were spheres only. Notably, the number of microadenoma-like structures from *fbxw7*^ΔG^ mutant organoids significantly decreased in response to 5-FU (Figure 3d,e and Supplementary Video S2). These findings together suggest that 5-FU-induced DNA damages may cause a reduction in cellular proliferation resulting in morphological changes within the mini-gut structure. In this regard, as the proliferative compartment is predominantly in the crypt-like domains, its impairment may lead to spherical mini-guts. These results support the hypothesis that the organoids can be used as *ex vivo* tools for investigating drug resistance.

### Fbxw7 loss counteracts organoids differentiation induced by 5-FU

**Figure 4:**
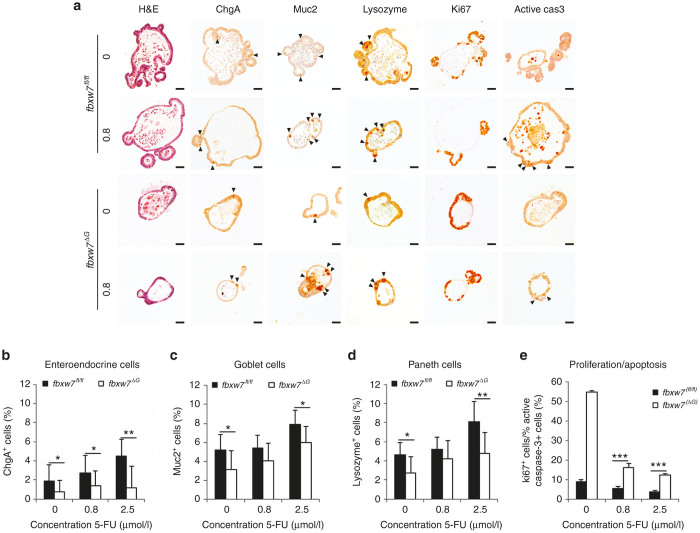
Fbxw7 loss impairs 5-FU-induced differentiation and, thus, apoptosis. (**a**) H&E staining and immunohistochemistry (IHC) analysis of enteroendocrine cells positive for chromogranine A (chgA), goblet cells positive for mucine (muc2), Paneth cells positive for lysozyme (lys), cycling cells (Ki67), and apoptotic cells positive for active caspase 3 (active cas 3) on *fbxw7*^*fl/fl*^ and *fbxw7*^ΔG^ mini-guts following incubation with 0.8 μmol/l 5-FU. Black arrowheads show positively stained cells. Scale bars, 50 μm. (**b–d**) Histograms show percentage of terminally differentiated cells and ratio between cycling cells and apoptotic cells (**e**) following IHC analyses. The relative number of positively stained cells in 50-untreated organoids was counted for each genotype (*fbxw*7^fl/fl^ versus *fbxw*7^ΔG^ mini-guts) and in 30-treated organoids for each dose of 5-FU and presented as % of positively stained cells to the total number of cells (*P < 0.05, **P < 0.01, ***P < 0.001).

**Figure 5:**
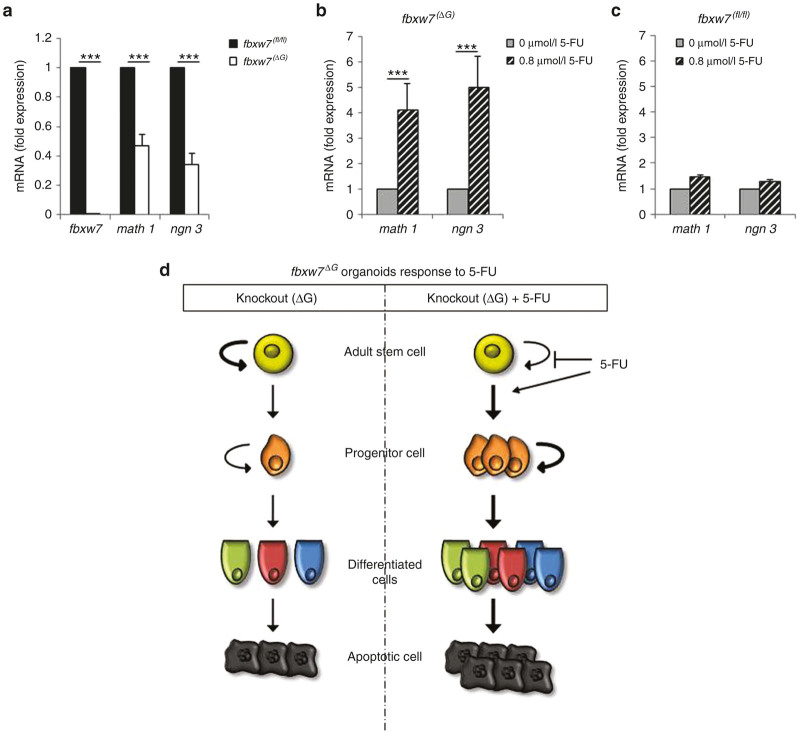
5-FU treatment promotes secretory progenitor cells in absence of Fbxw7. (**a**) *fbxw7*, *math1*, and *ngn3* expression was analyzed by quantitative real-time polymerase chain reaction (qRT-PCR) in *fbxw7*^ΔG^ mini-guts at day 7 of growth. Expression level was first normalized to β-actin and then to *fbxw7*^*fl/fl*^ mini-guts. (**b,c**) *math1* and *ngn3* expression was analyzed by qRT-PCR in *fbxw7*^*fl/fl*^ and *fbxw7*^ΔG^ mini-guts after 72 hours treatment with 0.8 μmol/l 5-FU. Expression was first normalized to β-actin and then to the corresponding untreated mini-guts (*P < 0.05, **P < 0.01, ***P < 0.001). (**d**) Schematic shows 5-FU induction of terminal differentiation and apoptosis in *fbxw7*^ΔG^ mini-guts.

To examine the effects of 5-FU on proliferation and differentiation, surviving organoids were fixed, embedded in paraffin, and processed for immunohistochemistry. As outlined above, a treatment with 7.5 μmol/l 5-FU induced cell death in almost but not all wild-type *fbxw7*^*fl/fl*^ organoids, and very few remaining live organoids formed spheres (Figure 3e), therefore we used the surviving organoids treated with 2.5 and 0.8 μmol/l 5-FU. Previous studies reported that Fbxw7 loss in murine intestinal epithelium resulted a reduction in secretory cells together with an increase in actively cycling cells within the crypts.^24,25^ Consistent with this, by comparing untreated samples, *fbxw7*^ΔG^ mini-guts exhibited a significantly lower percentage of secretory cells such as enteroendocrine cells positively stained for Chromogranin A (chg-A) (Figure 4a,b), goblet cells stained for mucin 2 (muc2) (Figure 4a,c) and Paneth cells stained for lysozyme (Figure 4a,d). Moreover, we examined whether there was correlation between Ki-67 proliferative activity and apoptosis in treated organoids. In line with previous studies of mouse models,^24–26^ we observed that the ratio between proliferative cells (Ki-67 positive) and apoptotic cells (active caspase 3 positive) was higher in mutant mini-guts (Figure 4a,e). Interestingly, 5-FU treatment induced goblet, Paneth, and enteroendocrine cells differentiation in a concentration-dependent manner (Figure 4a–d), indicating that 5-FU improves organoid peculiar structure by rescuing the cellular differentiation driven by loss of *fbxw*7. In addition, consistent with *fbxw7*^ΔG^ mice,^24,25^ expression of secretory progenitor markers *math-*1 (mouse atonal homologue-1) and *ngn-*3 (neurogenin-3) were also significantly repressed in organoids lacking of *fbxw*7 (Figure 5a). Interestingly, following 5-FU treatment, *math1* and *ngn3* expression was significantly increased about fourfolds in *fbxw7*^ΔG^ mini-guts (Figure 5b), in contrast with a slight increase in *fbxw7*^*fl/fl*^ mini-guts (Figure 5c). These data indicate that 5-FU promotes terminal differentiation and apoptosis in normal intestinal epithelium, potentially by triggering the DNA damage/repair pathway. Also, CRC harboring *FBXW7* mutations may acquire drug resistance via induction of proliferative progenitor cells and impairment of their terminal differentiation in colon/intestine.

## Discussion

Murine intestinal *fbxw*7-deletion blocks differentiation and drives hyperproliferation in the intestine.^24,25,28^ *In vitro* studies also reported that loss of FBXW7 function leads to the acquisition of drug resistance in CRC cell lines, through the induction of an epithelial-mesenchymal transition (EMT) pathway,^32^ and/or alteration of MCL1,^40^ mTOR,^41^ and TP53 status.^30^ In this study, to demonstrate that intestinal organoids are a useful and physiologically relevant model system to study the role of Fbxw7 in cancer biology and drug resistance, we have cultured organoids in the presence and absence of 5-FU. Due to their comparable structure and behavior,^12,38^ treated and untreated organoids (seeded initially with the same number of crypts) are analyzed across several key parameters over time; integrity, morphology, survival and apoptosis, and stained for various intestinal cell lineages. Here, we describe that *fbxw7*^ΔG^ organoids exhibited higher proliferative activity, thus driving faster maturation of the enterospheres into enteroids and, ultimately microadenoma-like structures, which disseminated cells outside the mini-gut lumen in a burst (Figure 2c and Supplementary Video S1).

Consistent with CRC cell lines, we further confirmed that Fbxw7 deficiency confers higher tolerance to 5-FU, a DNA damaging agent; the IC_50_ of *fbxw7* mutant mini-guts was significantly higher than controls (5.7 versus 2.6 μmol/l 5-FU, respectively) (Figure 3c). Analyzing the complex phenotypical changes in organoid culture, 5-FU treatment induced the transformation of the common crypt-villus morphology (enteroids) into cystic structures (spheres), in a dose-dependent manner (Figure 3d–f). Likewise the loss of Fbxw7-induced cellular transformation, the transformation of enteroids into spheres was also found in *Apc*-inactivated intestinal organoids,^42^ a key component of the Wnt/β-catenin signaling pathway in regulating intestinal epithelial homeostasis and the early stages of colorectal carcinogenesis.^43^ As predicted, the number of microadenoma-like mini-guts significantly decreased after 5-FU treatment (Figure 3e,f). Fbxw7-depleted mini-guts, showed an active proliferative compartment (Figure 4e) along with reduction of secretory progenitor cells (Figure 5a). According to these, loss of Fbxw7 leads to impaired intestinal terminal differentiation and therefore *fbxw*7^ΔG^ organoids appears smaller than *fbxw*7^fl/fl^ organoids. On the other hand, due to an early increased rate of proliferation, most of *fbxw*7^ΔG^ organoids quickly enlarged and exploded. Consequently, *fbxw*7^ΔG^ organoids were not homogeneous populations to quantify and compare their size with *fbxw*7^fl/fl^ organoids.

Our experiments do not point to molecular mechanism underneath FBXW7-rendered resistance to 5-FU. The role of FBXW7 in drug resistance is just beginning to emerge. Based on our own and recent published studies, there is a strong link between FBXW7 and cell cycle regulation as well as the EMT-mediated drug resistance in cancer cells.^30,32,40,44,45^ However, beyond the scope of this current study and the importance of given data here and our published and unpublished data on links between EMT and FBXW7, more recently, we have initiated a study using different approaches to explore molecular mechanism(s) underneath FBXW7-rendered resistance to DNA-damage agents including 5-FU in CRC, organoid, and animal models. Our current analysis however highlights significant downregulation of the cyclin-dependent kinase inhibitor p21 in *fbxw*7^ΔG^ organoids while the p53 related transcription factor p63 expression appeared to be not significantly changed. 5-FU led to the induction of p21 and p63 in wild-type *fbxw*7^fl/fl^ organoids but in *fbxw*7^ΔG^ organoids induced p63 and not p21 (Supplementary Figure S3a–c). The differential expression of p53 related genes further support the hypothesis of an existing relationship between mutated-Fbxw7 and p53 in response to drug treatment.^30^ Previously published studies showed that p53 upregulates Fbxw7^27,46^ whereas it represses ZEB family transcriptional regulators (ZEB1 and ZEB2), implicated in EMT.^47^

These findings clearly indicated that the percentage of enteroendocrine cells, goblet cells and Paneth cells were increased along with a reduction of cycling versus apoptotic cell ratio following 5-FU-mediated DNA damage. Fbxw7 depletion was found to influence cell commitment and proliferation in murine intestinal epithelium via activation of c-Jun and Notch signaling pathways.^24,25,28^ However, since the amount of proteins available for extraction from 5-FU-treated organoids, it is difficult to analyze how 5-FU modulates *math*1 and *ngn*3 in organoids. Previously published reports indicated that intestinal Notch activation upregulates Hes-1 and consequently, represses the transcription of *math*1 and *ngn*3.^48,49^ Therefore, protein extracts were isolated from untreated and 5-FU treated HCT116 and DLD-1 cell lines with FBXW7^(−/−)^ and FBXW7^(+/+)^ alleles and Western blotted for active NICD1 antibody, as a FBXW7 target for degradation.^50^ These observation further support that expression level of active NICD1 is increased in FBXW7^(−/−)^ cells while decreased in both FBXW7^(+/+)^ and FBXW7^(−/−)^ cells treated with 5-FU (Supplementary Figure S4a). We have also studied the relative mRNA expression of *MATH*1 and *NGN*3 genes by quantitative real-time polymerase chain reaction in CRC cell lines. Unfortunately with both Cyber green and TaqMan assays, *C*_t_ values of *MATH*1 and *NGN*3 were >32 (representing even higher *C*_t_ value than *FBXW*7 gene in K/O cells). We have additionally increased the amount of cDNA which had also no effect on *C*_t_ value (Supplementary Figure S4c). These suggest that *MATH*1 and *NGN*3 are very low expressed genes in these cells and may not be reliable to calculate their relative gene expression for the purpose of the experiment. In addition, terminal differentiation of the enteroendocrine producing cells has been shown by coordinating transcription of the secretin gene with cell cycle arrest.^51,52^ Hence, the modulation of *Math*1 and *ngn*3 by 5-FU could also be linked to the changes of cyclin-dependent kinase inhibitor p21 level as outlined above (Supplementary Figure S3b,c). However, further studies should be carried out in future, to further precisely explore these possibilities via loss and gain of functional analysis. Progenitor cells, abundant in *fbxw7*^ΔG^ mini-guts, may drive tumor recurrences and/or metastasis post-treatment. However, contrary to the organoid model, the *fbxw7*^ΔG^ mice alone did not promote intestinal tumorigenesis up to 11–12 months of age,^24,25^ whereas we predict that this genotype causes sufficient functional rearrangement to promote/recapitulate human tumorigenesis. Nevertheless, the organoid culture method allows a specific analysis of epithelial components of the intestine/colon and therefore these changes in morphology suggest an alteration between proliferative and differentiating compartments, induced independently of intestinal microenvironment.

We conclude that Fbxw7-mutated cancer cells are highly proliferative and resistant to the terminal differentiation and subsequent cell death that is normally triggered by chemotherapeutic DNA damage (Figure 5d). Furthermore, DNA damaging agents could cause mutations in cycling cells promoting the acquisition of prosurvival properties in drug-resistant cancer cells.^53,54^ Our findings suggest that a differentiation therapy may be one strategy to validate current chemotherapeutic drugs against the FBXW7-mutated CRCs. These results support the hypothesis that human mini-guts derived from cancer patients,^4^ and/or the modeling of cancer mutations in human intestinal/colon organoids using new tools for gene manipulation,^3,5^ may allow both *ex vivo* and *in vivo* experimental approaches investigating drug resistance, drug screening, and preclinical toxicological studies in molecularly targeted systems that mimic endogenous cancer driver mutations and eventually design efficacious cancer-specific drugs.

## Materials and Methods

### Mouse lines

Both *fbxw*7^fl/fl^ and *fbxw*7^∆G^ were described previously.^24^ Only same sex littermates of 4–6-week mice were used. Mice were housed and bred in a pathogen-free transgenic animal facility of the Biomedical Service Unit and all procedures complied with regulations and guidelines of the Biomedical Service Unit, University of Nottingham.

### Small intestine crypt isolation and organoid culture

Small intestine was resected from *fbxw7*^*fl/fl*^ and *fbxw7*^ΔG^ mice.^7,24^ Intestine was repeatedly washed with cold phosphate-buffered saline, then longitudinally opened and sectioned in small fragments. Crypts were released from the epithelium by incubation with 3 mmol/l ethylenediaminetetraacetic acid phosphate-buffered saline for 35 minutes at 4 °C. Following counting and isolation by centrifuge, crypts were resuspended with Matrigel (BD Biosciences, Oxford, UK), seeded into a 48-well plate and fed with advanced Dulbecco’s modified eagle medium/F-12 medium containing; N2 supplement (Invitrogen, Warrington, UK), B27 supplement (Invitrogen), 1 mmol/l N-acetylcysteine (Sigma-Aldrich, Dorset, UK), 50 ng/ml murine recombinant epidermal growth factor (Invitrogen), 100 ng/ml Noggin (Peprotech, London, UK), and 1 μg/ml R-Spondin1.^7^ 0.8, 2.5, and 7.5 μmol/l 5-FU (Tocris Bioscience, Bristol, UK) was administered at day 3 or day 4 of growth respectively to *fbxw7*^ΔG^ and *fbxw7*^fl/fl^ mini-guts, for viability and morphological analyses in response to drug treatment. Organoid were also monitored with integrated optics for phase-contrast imaging and machine vision technology in a humidified, 5% CO2 atmosphere at 37 °C for 5 days (Cell-IQ, CM Technologies Oy; www.c-mtechnologies.com). Media replaced every 48 hours and images captured automatically per well at half an hour intervals. Cell-IQ analyzer software was used according to manufacturer’s instructions.

### Cell culture, transfection, and western blotting analysis

Human colon cancer HCT116 and DLD-1 cells were fed with RPMI-1640 medium supplemented with 10% fetal bovine serum (Sigma-Aldrich) and 2 mmol/l L-glutamine (Life Technologies, Warrington, UK). Transfection of CRC cell lines with DNA plasmids was performed via Lipofectamine 3000 (Invitrogen) transfection reagent following the manufacturer’s instructions. RIPA lysis buffer supplemented with protease inhibitor cocktail was used to extract the protein content from both live and dead cells. Equal amount of total proteins was denaturized, loaded into a 10% sodium dodecyl sulfate-polyacrylamide gradient gel for separation and then transferred overnight to polyvinylidene fluoride membranes. Membranes were blocked with 3% bovine serum albumin in Tris-buffered saline and probed with different antibodies as indicated as previously described.^30^

### Cytotoxicity assay and colony-forming efficiency assay

Human colon cancer HCT116 and DLD-1 cells were fed with RPMI-1640 medium supplemented with 10% fetal bovine serum (Sigma-Aldrich) and 2 mmol/l L-glutamine (Life Technologies). 3,000 cells/well were seeded for cytotoxicity assay into 96-well plate in triplicate, synchronized by serum-starvation, and incubated with increasing concentrations of 5-FU for 72 hours. Cells were fixed with 6% trichloroacetic acid for 1 hour at 4 °C and then washed with water. 0Air dried plates were stained with 0.4% (w/v) sulforhodamine B in acetic acid for 20 minutes at room temperature and washed five times with 1% acetic acid. 10 mmol/l Trizma base was added and the absorbance read at 540 nm.^30^

To perform colony-forming efficiency assay, 500 cells (HCT116) were seeded into a six-well plate in triplicate. Then, 0, 2.5, and/or 7.5 μmol/l 5-FU was administered prior to cell-doubling, to reduce survival advantages associated to different proliferative capabilities. 72 hours after, 5-FU containing medium was replaced with a complete culture medium. Colonies were fixed with 4% paraformaldehyde for 20 minutes at room temperature and stained with 0.1% crystal violet for the following procedures to count number of colonies using Image J software.^30^

### Immunohistochemistry

Organoids were fixed in 4% paraformaldehyde overnight at 4 °C and resuspended in 2% low-melting agarose prior to paraffin embedding. 4-µm sections were cut and processed for immunohistochemistry analysis or Hematoxylin and Eosin (H&E) staining as previously reported.^24^ The following primary antibodies were used: active caspase 3 (R&D systems, Abingdon, UK), Chromogranin A and Ki-67 (Abcam, Cambridge, UK), Mucin 2 (Santa Cruz Biotechnology, Heidelberg, Germany), and Lysozyme (Dako, Cambridge, UK).

### Quantitative real-time polymerase chain reaction analysis

To dissociate the Matrigel, organoids were incubated with cell recovery solution (Corning, Corning, NY) for 3 hours in cold room. Total RNA was separated by using TRIZOL reagent (Sigma-Aldrich) and precipitated following manufacturer’s instructions. cDNA synthesis was obtained by using PrimeScript Reverse Transcriptase (TAKARA, Saint-Germain-en-Laye, France) according to the manufacturer’s instructions. Quantitative real-time polymerase chain reaction was performed based on the incorporation of SYBR green (Life Technologies). Primers^24^ used are listed in Supplementary Table S1.

### Statistics

All statistical analyses were evaluated with Student’s *t*-tests and the Mann-Whitney *U*-test, as appropriate. A *P* value of < 0.05 was considered statistically significant, where experiments were repeated at least two and three times in triplicates, respectively.
