# Supplementary material for: Fbxw7-associated drug resistance is reversed by induction of terminal differentiation in murine intestinal organoid culture
Source: Mol Ther Methods Clin Dev. 2016 Apr 13;3:16024–. doi: 10.1038/mtm.2016.24 (PMC4830362; doi:10.1038/mtm.2016.24)
Supplement: Supplementary Information [file mtm201624-s1.zip › mtm-00269-s01.docx]

**SUPPLEMENTARY MATERIALS**

This file contains Supplementary Figures 1-4, Supplementary Tables 1 & Supplemental Videos 1 & 2.

# Fbxw7-associated Drug Resistance is reversed by Induction of Terminal Differentiation in Murine Intestinal Organoid Culture

Federica Lorenzi^1^, Roya Babaei-Jadidi^1^, Jonathan Sheard^2^, Bradley Spencer-Dene^3^ & Abdolrahman S. Nateri^1*^

^1^ Cancer Genetics and Stem Cell Group, Cancer Biology Unit, Division of Cancer and Stem Cells, School of Medicine, University of Nottingham, Nottingham NG7 2UH, UK

^2^ CM Technologies Oy I, Institute for Biomedical Technology, University of Tampere, Biokatu 12, 33520 Tampere I, Finland

^3^ Experimental Pathology Laboratory, Cancer Research UK London Research Institute, London WC2A 3PX, England, UK

* Corresponding: Abdolrahman S. Nateri, Ph.D. Email: a.nateri@nottingham.ac.uk

**Supplementary Figure S1. Loss of FBXW7 confers 5-FU resistance to human colon cancer DLD-1 cells. (a)** RT-PCR shows *FBXW7* status in DLD-1^FBXW7(+/+)^ and DLD-1^FBXW7(-/-)^ cell lines. Reverse primer was designed to target the deleted exon 5 of *FBXW7*. **(b, c)** Cytotoxicity assay (SRB assay) of 5-FU in both DLD-1^FBXW7(+/+)^ and DLD-1^FBXW7(-/-)^ cell lines. Vehicle (DMSO) and ten doses of 5-FU (from 0.05 μM to 100 μM) were incubated for 72 hours following cell synchronization. **(d, e)** SRB assay of 5-FU of transiently transfected DLD-1^FBXW7(-/-)^ cells with pcDNA3 (control) or FLAG-FBXW7α plasmids. Table reports obtained IC_50_ values. Experiments were performed in triplicate and repeated at least in three independent occasions. Data are mean ± SEM (**P*<0.05, ***P*<0.01, ****P*<0.001). **(f)** FLAG-tagged version of FBXW7 (FLAG-FBXW7) protein was overexpressed in HCT116^FBXW7(-/-)^and DLD-1^FBXW7(-/-)^cells, and lysates were subjected to immunoblotting with anti-FLAG. β-actin was used as a loading control.

**Supplementary Figure S2. *villin*-Cre mediated recombination of intestinal *fbxw*7.**

**(a)** Representation of the *lox*P flanked exon 5 of *fbxw*7 (*fbxw*7^fl/fl^), Cre recombinase is regulated by *villin*-promoter (*villin*-Cre) and the followed excision of *fbxw*7 exon 5 limited to the gut epithelium (*fbxw*7^ΔG^). Arrows show the complementary sites for PCR primers. **(b)** PCR analysis of genomic DNA from *fbxw*7^fl/fl^ and *fbxw*7^ΔG^ mice.

**Supplementary Figure S3. *fbxw7*^ΔG^ organoids down-regulate the cyclin-dependent kinase inhibitor, p21. (a)** *p21* and *p63* expression levels were measured by qRT-PCR in *fbxw7*^ΔG^ mini-guts at day 4 of growth. Expression level was first normalized to β-actin and then to *fbxw7^fl/fl^* mini-guts. **(b, c)** *p21* and *trp63* expression was examined by qRT-PCR in *fbxw7*^ΔG^ and *fbxw7^fl/fl^* mini-guts after 72 hours treatment with 0.8 μM 5-FU. Expression was first normalized to β-actin and then to the corresponding untreated mini-guts (**P*<0.05, ***P*<0.01, ****P*<0.001).

**Supplementary Figure S4. 5-FU treatment induced impairment of Notch signalling pathway in HCT116 and DLD-1 cell lines. (a)** Western blotting analysis of NICD1 protein level in HCT116 and DLD-1 cells. Total proteins were isolated after 72 hours incubation with vehicle (DMSO) and 7.5 μM 5-FU (IC_50_ of FBXW7-wild type cell lines). β-actin was used as loading control. **(b)** *FBXW7* expression was analysed by qRT-PCR in HCT116 and DLD-1 cell lines. Expression was first normalized to β-actin and then to the corresponding untreated mini-guts (**P*<0.05, ***P*<0.01, ****P*<0.001).  **(c)** Amplification curves of *FBXW*7, *MATH*1 and *NGN*3 expression levels of HCT116^FBXW7(+/+)^, HCT116^FBXW7(-/-)^, DLD-1^FBXW7(+/+)^ and DLD-1^FBXW7(-/-)^ cell lines were examined via qRT-PCR following incubation with vehicle (DMSO) and 7.5 μM 5-FU as outlined above.

**Supplementary Table S1. Primer sequences for real time RT-PCR, RT-PCR and PCR analyses.**

**Supplementary Video S1. The peculiar morphology of *fbxw*7^ΔG^ organoids (Cre +Ve, Control)**

**Supplementary Video S2. *fbxw*7^ΔG^ organoids treatment with 5-FU (Cre +Ve, Drug)**
