# Supplementary figures and images for: Fbxw7-associated drug resistance is reversed by induction of terminal differentiation in murine intestinal organoid culture
Source: Mol Ther Methods Clin Dev. 2016 Apr 13;3:16024–. doi: 10.1038/mtm.2016.24 (PMC4830362; doi:10.1038/mtm.2016.24)

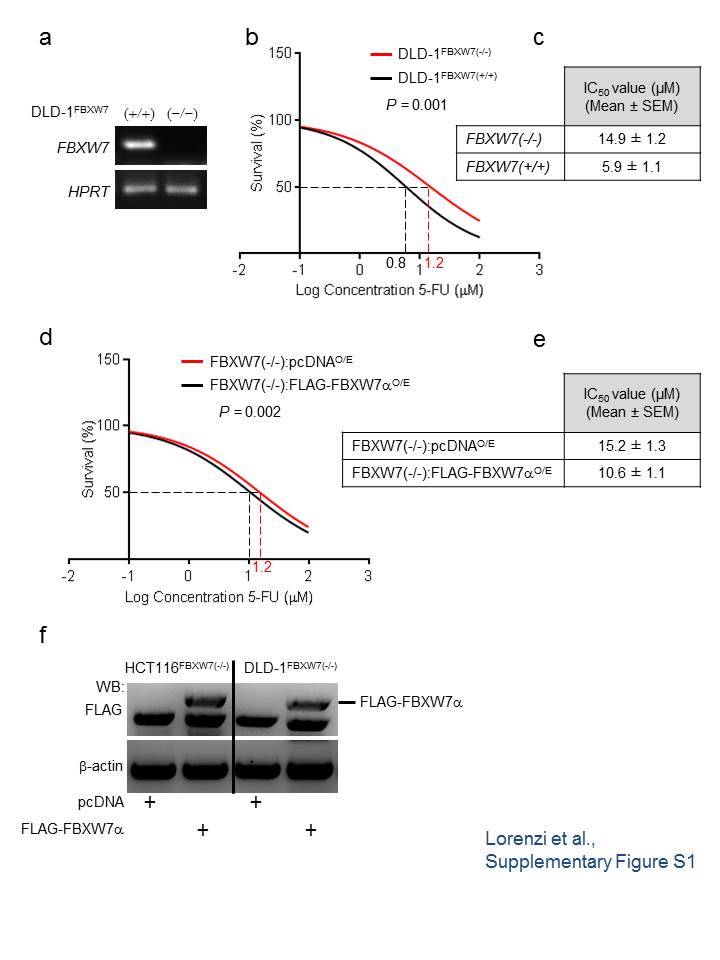

Supplement: Supplementary Information [file mtm201624-s1.zip › mtm-00269-s02.jpg]

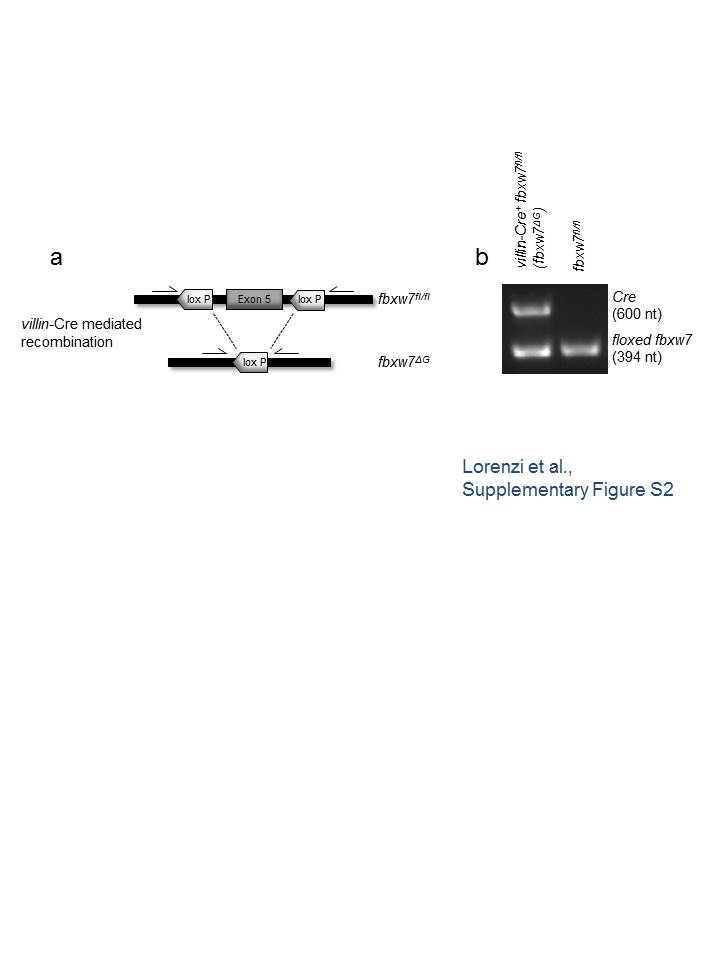

Supplement: Supplementary Information [file mtm201624-s1.zip › mtm-00269-s03.jpg]

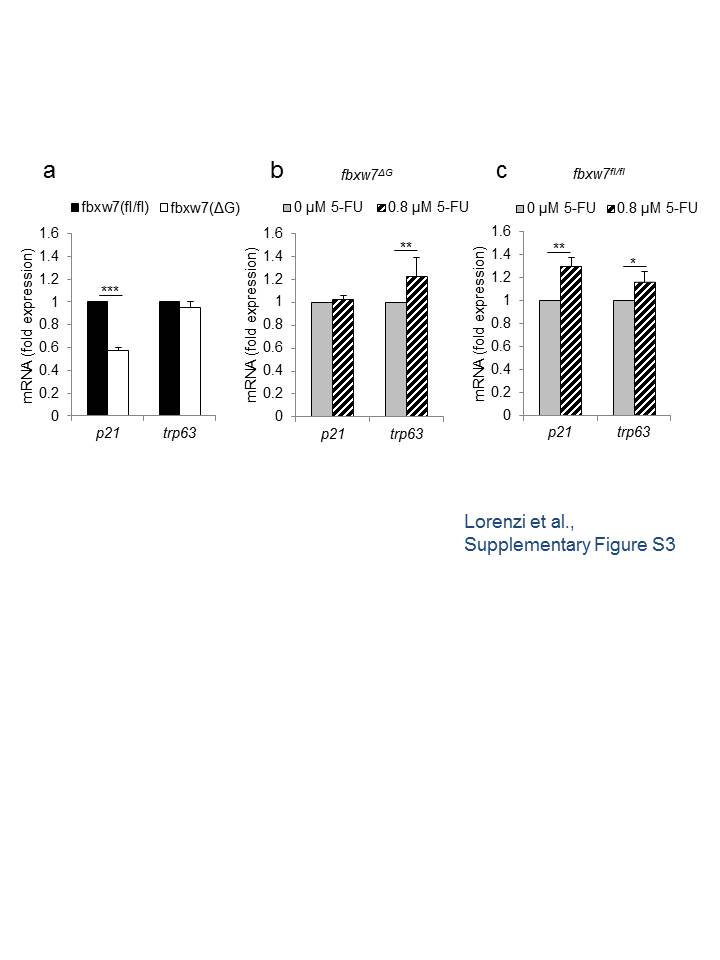

Supplement: Supplementary Information [file mtm201624-s1.zip › mtm-00269-s04.jpg]

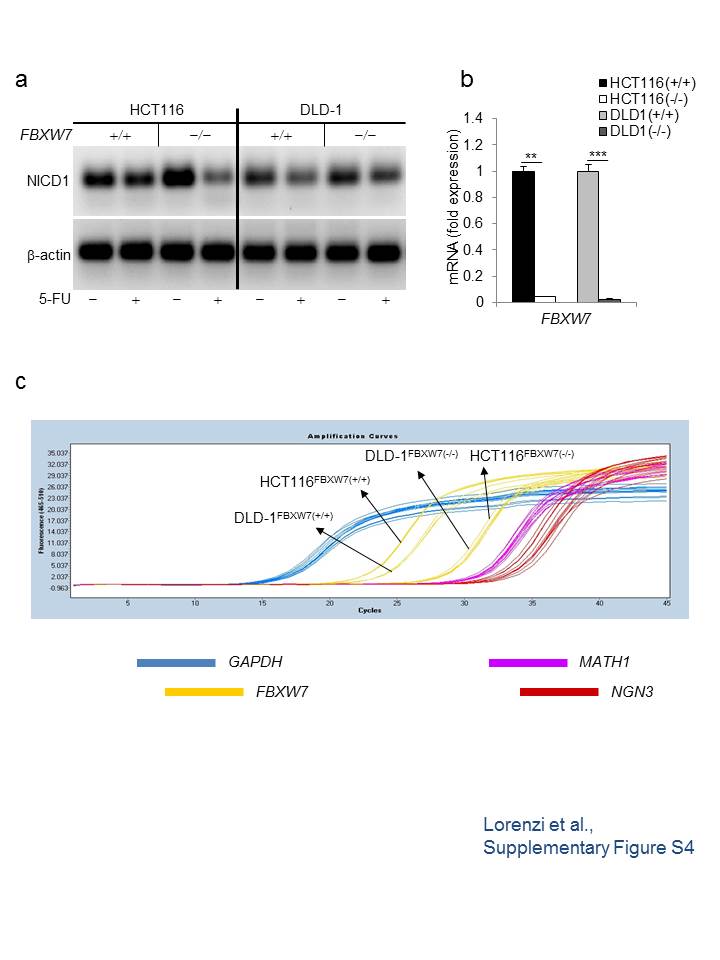

Supplement: Supplementary Information [file mtm201624-s1.zip › mtm-00269-s05.jpg]

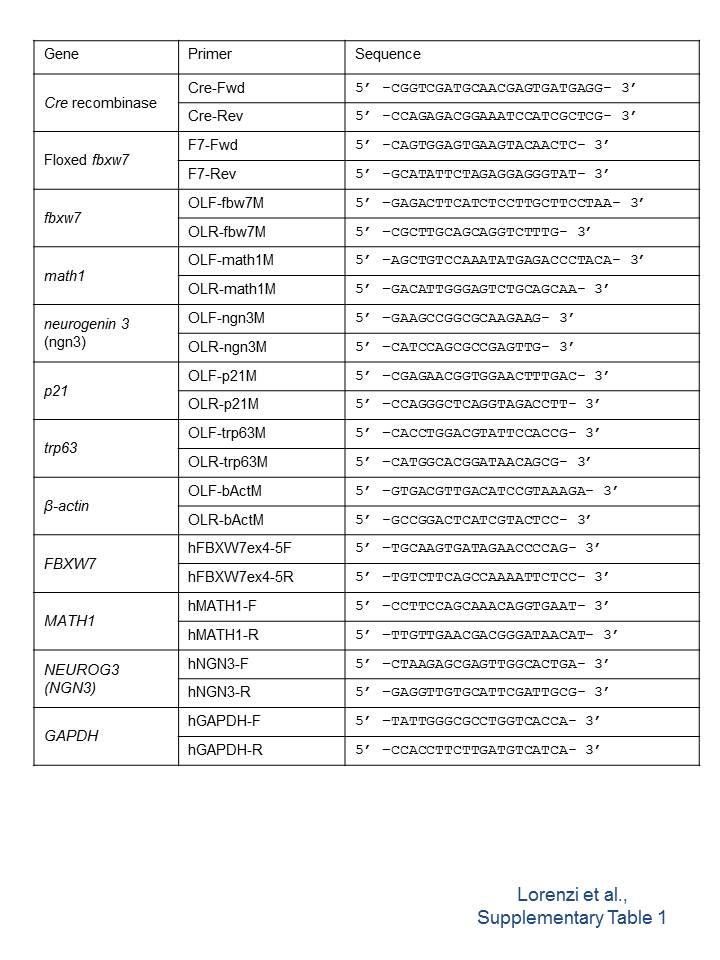

Supplement: Supplementary Information [file mtm201624-s1.zip › mtm-00269-s06.jpg]
